# How often is each gene mutated within the cancer patient population?

**DOI:** 10.1080/23723556.2022.2065176

**Published:** 2022-05-01

**Authors:** Gaurav Mendiratta, Michael K. Jones, Edward C. Stites

**Affiliations:** Integrative Biology Laboratory, Salk Institute for Biological Studies, La Jolla, CA, USA

**Keywords:** Cancer gene mutations, gene mutations epidemiology, mutations rate, cancer mutations incidence, cancer types ROSETTA

## Abstract

Genome sequenced samples from cancer patients helped identify roles of different mutation types and enabled targeted therapy development. However, critical questions like what are the gene mutation rates among the patients? or what genes are most commonly mutated, pan-cancer? have only been recently answered. Here, we highlight this recent advance.

Accessibility to genomic data as a result of technological advances gave rise to a better understanding of the roles that specific gene mutations play in the development and progression of specific types of cancer, as well as for cancer in general. As a consequence, the advent of cancer genomics presented the welcomed promise of both increased understanding of the cellular and molecular mechanisms which underlie the causes of cancer; and, importantly, the information identifying targets for personalized therapies that could substantially reduce the mortality and morbidity burdens of this devastating disease. However, some very basic questions cannot be addressed by these data, such as: Within the cancer patient population, how often is any given gene likely to be mutated? What are the most commonly mutated genes across all cancer patients? Presumably, targets for personalized therapies could be better prioritized with a better understanding of their true incidence in the patient population.

Currently available pooled and pan-cancer resources^1–4^ cannot answer the aforementioned questions because the number of cancer samples in those resources are not proportionate to the actual incidence of those cancers (Figure 1). To overcome this problem, we introduced a framework that performs a post-hoc analysis of available cancer genomic data and integrates the sequencing data with cancer epidemiological data to remove some of the sample bias from the genomic sequencing studies. This allowed us to calculate mutation frequency estimates that better represent the cancer patient population of the United States.^5^ Mathematically, we calculated the “weighted average” by multiplying the rate of each gene mutation within each form of cancer by the proportion of all cancers that is accounted for by that specific form of cancer. However, accomplishing this undertaking was complicated by the fact that cancer genomics and cancer epidemiology utilize distinct naming systems for the same type of cancer. To overcome this, we created a new scheme for the reclassification of sequencing and epidemiological tumor type annotations (ROSETTA). With ROSETTA, 93% of sequenced cancer samples were mapped to an appropriate equivalent cancer type from the epidemiological data obtained from NCI SEER survey.

**Figure 1.**
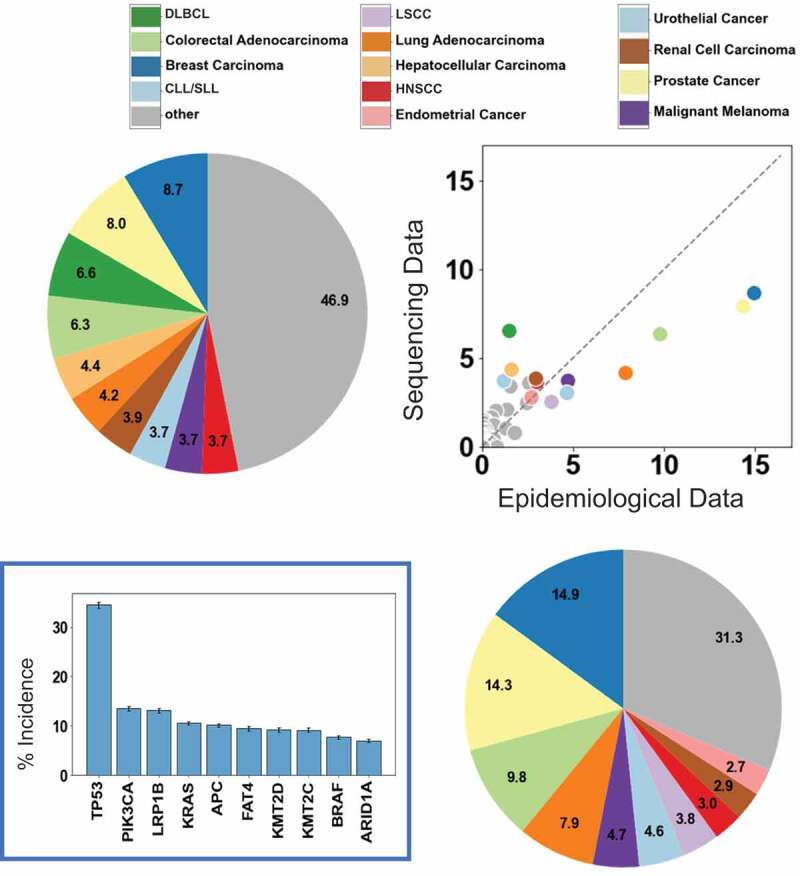
Epidemiologically re-weighted gene mutation rates. The scatter plot compares the (ROSETTA) cancer types data from 19,181 sequenced patient samples with ~7 million cases from the epidemiological SEER survey. The pie charts present the distribution of ROSETTA cancer types from sequenced samples (top left) and from SEER (bottom right). (Inset) the top ten incident cancer types are color coded. Reweighted pan-cancer incidence rates (%) in the US population are shown for commonly mutated genes.

Abbreviations: DLBCL – Diffuse large B cell lymphoma; LSCC - Lung Squamous Cell Carcinoma; CLL/SLL - Chronic lymphocytic leukemia/small lymphocytic leukemia; HNSCC – Head Neck Squamous Cell Carcinoma.

We took exome data from 139 different studies. These studies included 19,181 different samples. We processed these data with ROSETTA. Importantly, of the 22,730 different samples, we found 3,549 were redundant (same sample included in one or more studies or longitudinal samples for the same patient) and we excluded redundant/repeat samples from our analysis. We also excluded sequenced cell lines and xenografts due to the potential for specific mutations to influence the ability of a cancer cell to propagate in either condition. We then processed cancer epidemiology data from more than 7 million patients through ROSETTA to obtain our estimates for the proportion of all cancers due to each category of cancer. Once both types of data were processed, we computationally integrated the data. We also calculated 95% confidence intervals by generating Poisson-distributed in-silico genomic studies for each gene and ROSETTA cancer type, which were then processed through the reweighting pipeline. Software for our analysis is publicly available via (GitHub: https://github.com/GMendiratta/ROSETTA-for-Cancer-Mutations).

Our analysis yielded several surprising findings, including that oncogenic driver genes were far less common than previously thought (Figure 1). *PIK3CA* is found to be the most commonly mutated proto-oncogene, being mutated in 13% of cancers. This is notable, as *KRAS* is often stated to be the most commonly mutated proto-oncogene.^6^ Our study found *KRAS* mutated in 11% of all cancers as opposed to the 30% mutation rate often quoted, including in the recent literature.^7,8^ We also report that a major effector of Ras proteins, encoded by the gene *BRAF* has a mutation incidence of 8%, almost as common pan-cancer as *KRAS*.

One important implication of our study is that it highlights how cancer researchers have held inaccurate perceptions as to how commonly specific genes are mutated in cancer. The belief that *KRAS*, *NRAS*, and *HRAS* mutations are found in nearly one-third of all cancers helped motivate the creation of high-profile major research programs that focused on RAS^,9^ but our study found that only approximately 15% of all cancer patients have *KRAS*, *NRAS*, and/or *HRAS* mutation. It may be prudent to evaluate whether resources should be distributed to other high-mutation prevalence genes in a more equitable manner.

The mutant proteins encoded by mutated genes have been important drug targets for cancer. Our study suggests that there are few genes that are found mutated in a large fraction of cancer patients. However, more than 1.9 million new cancer diagnoses are projected for 2022^10^ and a gene found in 5% (or 1%) of patients would affect nearly 95,000 (or 19,000) patients every year! Our study finds 122 (incidence >5.00%) or 5,577 (incidence >1.00%) such genes exist. Thus, although our study suggests the road forward for personalized cancer medicine may be less direct, it also suggests that there are many possible paths forward.

## Figures and Tables

**Table:** 

NCI	National Cancer Institute
SEER	Surveillance, Epidemiology, and End Results
ROSETTA	Reclassification of sequencing and epidemiological tumor type annotations
PIK3CA	Phosphatidylinositol-4,5-bisphosphate 3-kinase, catalytic subunit alpha
RAS	Rat Sarcoma viral oncogenes (including KRAS, NRAS, NRAS)
KRAS	Kirsten RAS homolog
NRAS	Neuroblastoma RAS homolog
HRAS	Harvey RAS homolog
BRAF	Rapidly Accelerated Fibrosarcoma proto-oncogene homolog B

## References

[1] AACR Consortium, Project Genie. AACR project GENIE: powering precision medicine through an international consortium. Cancer Discov (AACR). 2017;7(8):818–3. doi:10.1158/2159-8290.CD-17-0151. PMC5611790 28572459

[2] Cerami E, Gao J, Dogrusoz U, Gross BE, Onur Sumer S, Arman Aksoy B, Jacobsen A, et al. The cBio cancer genomics portal: an open platform for exploring multidimensional cancer genomics data. AACR. 2012;2:401–404. 10.1158/2159-8290.CD-12-0095 PMC3956037 22588877

[3] Sondka Z, Bamford S, Cole CG, Ward SA, Dunham I, Forbes SA. The COSMIC cancer gene census: describing genetic dysfunction across all human cancers. Nat Rev Cancer (Nature Publishing Group). 2018;18(11):696–705. doi:10.1038/s41568-018-0060-1. PMC6450507 30293088

[4] Weinstein JN, Collisson EA, Mills GB, Shaw KR, Ozenberger BA, Ellrott K, Shmulevich I, Sander C, Stuart JM. The cancer genome atlas pan-cancer analysis project. Nat Genet (Nature Publishing Group). 2013;45(10):1113–1120. doi:10.1038/ng.2764. PMC3919969 24071849

[5] Mendiratta G, Ke E, Aziz M, Liarakos D, Tong M, Stites EC. Cancer gene mutation frequencies for the US population. Nat Commun (Nature Publishing Group). 2021;12:1–11. 10.1038/s41467-021-26213-y PMC8514428 34645806

[6] Ryan MB, Corcoran RB. Therapeutic strategies to target RAS-mutant cancers. Nat Rev Clin Oncol (Nature Publishing Group). 2018;15(11):709–720. doi:10.1038/s41571-018-0105-0. 30275515

[7] Venkatanarayan A, Liang J, Yen I, Shanahan F, Haley B, Phu L, Verschueren E, Hinkle TB, Kan D, Segal E, et al. CRAF dimerization with ARAF regulates KRAS-driven tumor growth. Cell Rep (Elsevier). 2022;38(6):110351. doi:10.1016/j.celrep.2022.110351. 35139374

[8] Merz V, Gaule M, Zecchetto C, Cavaliere A, Casalino S, Pesoni C, Contarelli S, et al. Targeting KRAS: the elephant in the room of epithelial cancers. Front Oncol (Frontiers). 2021;11:361. 10.3389/fonc.2021.638360 PMC7991835 33777798

[9] Stephen AG, Esposito D, Bagni RK, McCormick F. Dragging ras back in the ring. Cancer Cell (Elsevier). 2014;25(3):272–281. doi:10.1016/j.ccr.2014.02.017. 24651010

[10] Siegel RL, Miller KD, Fuchs HE, Jemal A. Cancer statistics, 2022. CA Cancer J Clin (Wiley Online Library). 2022.

